# Social, economic, political and health system and program determinants of child mortality reduction in China between 1990 and 2006: A systematic analysis

**DOI:** 10.7189/jogh.02.010405

**Published:** 2012-06

**Authors:** Xing Lin Feng, Evropi Theodoratou, Li Liu, Kit Yee Chan, David Hipgrave, Robert Scherpbier, Hana Brixi, Sufang Guo, Wen Chunmei, Mickey Chopra, Robert E. Black, Harry Campbell, Igor Rudan, Yan Guo

**Affiliations:** 1 Department of Health Policy and Administration, School of Public Health, Peking University, Beijing, China; 2 Centre for Population Health Sciences and Global Health Academy, University of Edinburgh Medical School, Edinburgh, Scotland, UK; 3 Department of International Health, Johns Hopkins Bloomberg School of Public Health, Baltimore, USA; 4 Nossal Institute for Global Health, University of Melbourne, Melbourne, Australia; 5 UNICEF Office for China, Beijing, PR China; 6 World Health Organization, Office of the WHO Representative in China, Beijing, PR China; 7 UNICEF Headquarters, New York, USA; * Joint first or senior authorship

## Abstract

**Background:**

Between 1990 and 2006, China reduced its under-five mortality rate (U5MR) from 64.6 to 20.6 per 1000 live births and achieved the fourth United Nation’s Millennium Development Goal nine years ahead of target. This study explores the contribution of social, economic and political determinants, health system and policy determinants, and health programmes and interventions to this success.

**Methods:**

For each of the years between 1990 and 2006, we obtained an estimate of U5MR for 30 Chinese provinces from the annual China Health Statistics Yearbook. For each year, we also obtained data describing the status of 8 social, 10 economic, 2 political, 9 health system and policy, and six health programmes and intervention indicators for each province. These government data are not of the same quality as some other health information sources in modern China, such as articles with primary research data available in Chinese National Knowledge Infrastructure (CNKI) and Wan Fang databases, or Chinese Maternal and Child Mortality Surveillance system. Still, the comparison of relative changes in underlying indicators with the undisputed strong general trend of childhood mortality reduction over 17 years should still capture the main effects at the macro-level. We used multivariate random effect regression models to determine the effect of 35 indicators individually and 5 constructs defined by factor analysis (reflecting effects of social, economic, political, health systems and policy, and health programmes) on the reduction of U5MR in China.

**Results:**

In the univariate regression applied with a one-year time lag, social determinants of health construct showed the strongest crude association with U5MR reduction (R^2^ = 0.74), followed by the constructs for health programmes and interventions (R^2^ = 0.65), economic (R^2^ = 0.47), political (R^2^ = 0.28) and health system and policy determinants (R^2^ = 0.26), respectively. Similarly, when multivariate regression was applied with a one-year time lag, the social determinants construct showed the strongest effect (beta = 11.79, *P* < 0.0001), followed by the construct for political factors (beta = 4.24, *P* < 0.0001) and health programmes and interventions (beta = −3.45, *P* < 0.0001). The 5 studied constructs accounted for about 80% of variability in U5MR reduction across provinces over the 17-year period.

**Conclusion:**

Vertical intervention programs, health systems strengthening or economic growth alone may all fail to achieve the desired reduction in child mortality when improvement of the key social determinants of health is lagging behind. To accelerate progress toward MDG4, low- and middle-income countries should undertake appropriate efforts to promote maternal education, reduce fertility rates, integrate minority populations and improve access to clean water and safe sanitation. A cross-sectoral approach seems most likely to have the greatest impact on U5MR.

Reduction of the under-five mortality rate (U5MR) has been recognized by the United Nations as one of the leading global priorities, and the fourth Millennium Development Goal (MDG4) calls on countries to reduce their U5MR by two-thirds from their 1990 baseline [1]. The latest Countdown Report finds only 19 of 68 target countries are on track to achieving this goal [2]. Evidence based guidance on the optimal mix of investments could greatly assist in accelerating progress.

Industrialized western countries achieved reductions in U5MR greater than 70% in the 30-year period between 1900 and 1930 [3-6], from baselines comparable to the rates observed in sub-Saharan African countries today [5,6]. This large decline has been attributed to economic development, improved diets and housing [7,8]. Economic progress alone, however, is not the answer; while there is clearly a correlation between U5MR and gross domestic product per capita (GDP) [9], there are many pairs of countries with 10-fold or greater difference in GDP but the same level of U5MR, and vice versa [10]. Analysis of more recent declines in child mortality have broadly identified several other key determinants of child survival, including maternal education [11-14], parental socio-economic status [15,16], public health expenditure and access to health services [14-18], sanitation and access to clean water and electricity [17], fertility rate [15,19], household income [15,19] and integration of minority population groups [14,20]. However, inconsistency and even contradiction among studies abounds and the interplay among these determinants and their relative importance in reducing U5MR remain unclear.

Most studies that have tried to identify the main drivers of U5MR reduction have relied on national-level data assembled through time series studies and indicators from nationally representative exercises such as Demographic Health Surveys (DHS) or Multi-Indicator Cluster Surveys (MICS) [11,12]. These studies were limited in their scope, the number of indicators that they used, the quality and quantity of the information available on mortality trends and the rigour of the analytic methods, thus limiting the inferences that can be drawn from them. The availability of annual child mortality data along with data related to a wide range of relevant determinants for each of 30 Chinese provinces over a 17-year period [21,22] provided the opportunity to conduct a more rigorous assessment of determinants of child mortality.

In the period between 1990 and 2006, China reduced its U5MR from 64.6 to 20.6 per 1000 live births, thus achieving MDG4 nine years ahead of schedule in a population of over 80 million under-fives [23]. This study explores the contribution of social, economic and political determinants, health system and policy determinants, and health programmes and interventions to this success using 35 indicators and provincial U5MRs from 30 Chinese provinces over the period 1990–2006.

## METHODS

### Data sources

**Figure 1:**
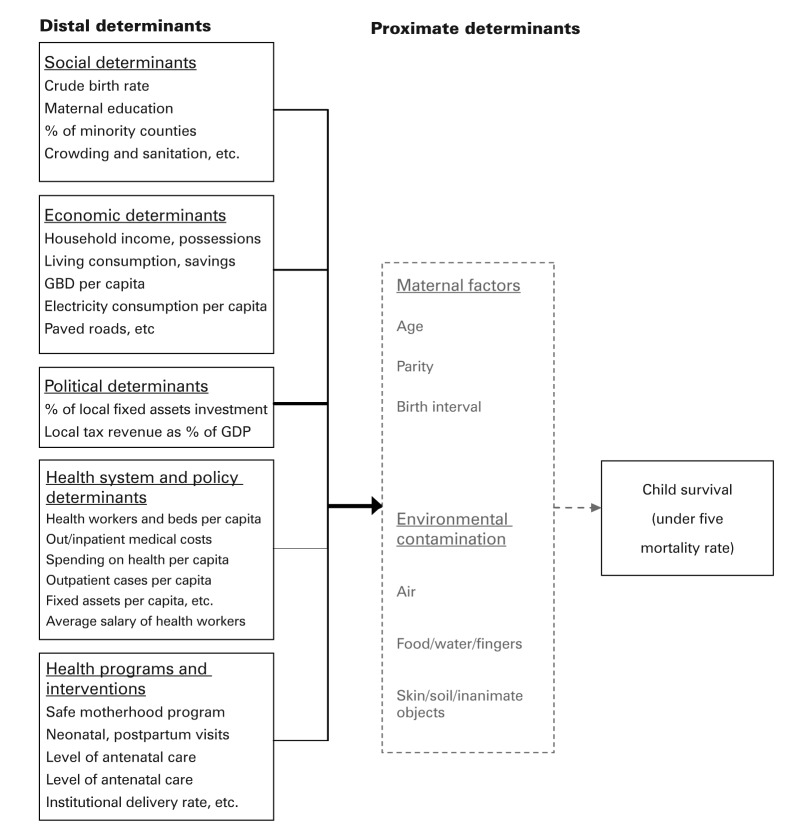
Conceptual framework of social, economic, political, and health system determinants of child survival. Adapted from Mosley and Chen (1984).

For the starting year (1990), we obtained U5MR data for 30 provinces, measured as the number of under-five deaths per 1000 live births, from the Chinese national report on neonatal, infant and under-five mortality [10,22]. We believe that those baseline rates are plausible because they were derived from a nation-wide neonatal, infant and under-five mortality rate study conducted in 1990 [22]. For each year between 1992 and 2006, we obtained an estimate of U5MR for the same 30 provinces from the China Health Statistics Yearbook (CHSY) [21]. We combined Chongqing and Sichuan Province for consistency across time, because Chongqing had been under the administration of Sichuan Province and became a Municipality directly under the Central Government in 1997. The CHSY reports province level U5MR estimates based on data from China’s Maternal and Child Health Annual Report system. This vital registration system collects information on births and maternal and child deaths at rural county and urban district level. A detailed description of the annual report system and its quality is available in recent publications [23-25]. The reliability of province-level U5MR estimates was much improved from 1996 onwards when the “Maternal and Infant Law” [26] was passed and the collection and management of the data were centralized by statisticians in the School of Public Health, Peking University. For further details about data sources and quality please see Online Supplementary Document(Online Supplementary Document) (table w1). Based on an explicit set of criteria (Online Supplementary Document(Online Supplementary Document), table w2), we decided to impute the data in the period 1991-1995 in the 16 provinces with inconsistent data during this period. In the other 14 provinces with plausible data, the missing U5MR data for 1991 were imputed by assuming a linear trend between 1990 and 1992. Overall, 416 (81.6%) data points for the U5MR outcome variable were based on the reported estimates and 94 (18.4%) were imputed because of concerns over data quality. **Figure 1** displays the trends of U5MR in each province between 1990 and 2006.

For each study year we also obtained province-level data on different social (n = 8), economic (n = 10), political (n = 2), health system and policy (n = 9) and health programmes and intervention (n = 6) indicators, available for each province and every year. The 20 social, political and economic indicators were extracted from the National and Provincial Statistics Yearbook (NPSY) [21]. Seven of the health system indicators were identified from the CHSY and the other seven were retrieved from the Health Finance Annual Report [27]. We also created a dummy variable indicating the coverage of China’s Safe Motherhood Program which was initiated in 2000 in selected high U5MR provinces [24]. A detailed description of the source, definition, and measurement unit of each indicator is provided in Online Supplementary Document(Online Supplementary Document) (tables w1 and w3).

The government data on province-level mortality and indicators are not of the same quality as some other health information sources in modern China, such as articles with primary research data available in CNKI and Wan Fang databases, or Chinese Maternal and Child Mortality Surveillance system, which were used in some of our recent high-profile publications [23-25]. Still, we believe that the comparison of relative changes in underlying indicators with the undisputed strong general trend of childhood mortality reduction over 17 years should still capture the main effects at the macro-level and should be useful for drawing very general conclusions.

### Statistical analysis

**Figure 2:**
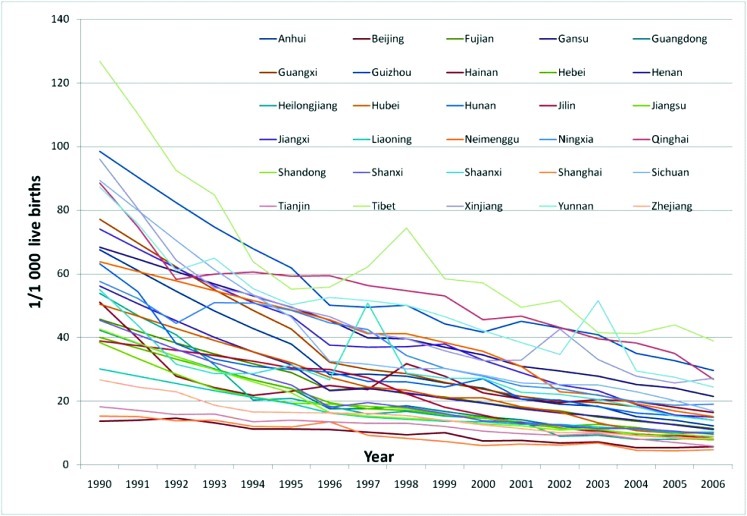
Trends of U5MR by provinces in China 1990-2006.

A detailed description of our step-wise approach to the analysis of these data is presented in the Online Supplementary Document(Online Supplementary Document) (table w2). We based our analysis on a conceptual framework that is adapted from the widely accepted Mosley and Chen child survival framework [28]. We conceptualized that distal determinants, including social, economic, political, health system and policy and health programs and interventions, act through a set of proximal determinants to affect child survival, as measured by U5MR. We took a reduced-form approach [29] to specifically examine the association between the 5 distal determinants and U5MR (see **Figure 2**).

Based on this conceptual framework, we first ran univariate and multivariate regression models to estimate the association between each of the 35 indicators and U5MR in each province (Online Supplementary Document(Online Supplementary Document), tables w4 and w5). We used a random effects linear regression model, taking into account the clustering of annual U5MR within each province. The indicators were standardized to facilitate comparison of the regression coefficients across indicators (see Online Supplementary Document(Online Supplementary Document), table w2, for details).

We then grouped the 35 indicators into 5 separate categories to capture the effects of social, economic, political, health system and policy, and health programmes and interventions determinants in each province. Factor analysis was conducted to extract the main variation from variables in each group. One factor was created per group to represent the majority proportion of common variation within that group. The 35 indicators were assigned to each of the 5 factors (‘constructs’) based on their maximum loadings on each factor, as shown in **Table 1**.

**Table 1:** Loading scores of determinants in their corresponding construct

Determinants	Health construct factors				
	**Health system and policy**	**Health program and intervention**	**Economic**	**Social**	**Political**
Number of health workers or doctors per 1000 population	0.4456				
Number of hospital beds per 1000 population	0.3828				
Outpatient medical costs per capita	0.9657				
Inpatient medical costs per capita	0.9719				
Total spending on health per capita	0.9905				
Public spending on health per capita	0.7712				
Number of outpatient cases per 1000 population	0.4979				
Fixed assets per capita	0.9482				
Average salary of health workers	0.8493				
Institutional delivery rate		0.7490			
Level of antenatal care		0.7953			
Level of postpartum visit		0.9010			
Level of neonatal visit by physician		0.7762			
Child care systematic management rate		0.7751			
Safe Motherhood Program indicator		0.0308			
Urban household income			0.9346		
Rural household income			0.9716		
Urban living consumption			0.9095		
Rural living consumption			0.9549		
Household possessions index			0.9277		
Bank savings per capita			0.9288		
GDP per capita			0.9710		
Electricity consumption per capita			0.7998		
Paved roads per square kilometres			0.7686		
Cargo turnover per capita			0.5743		
Percentage of autonomous ethnic minority counties				0.5605	
Illiteracy rate of women aged 15+				0.7103	
Crude birth rate				0.8481	
Urban household crowding				0.4663	
Rural household crowding				0.7781	
Percentage of household with clean water				−0.6631	
Hygienic toilet coverage				−0.7656	
Population density				−0.5710	
Proportion of local fixed assets investment					−0.7071
Local tax revenue as % of GDP					0.7071
**Proportion of common variation accounted**	0.7590	0.9000	0.9066	0.8414	0.6804

The 5 constructs, ie, the social, economic, political, health system and policy, and health programmes and interventions, were entered into the same random effects linear regression model described above. Univariate and multivariate regressions were again conducted to compare the unadjusted and adjusted associations between the 5 covariates and the province-level U5MR. To take into consideration possible time lags between changes in the 5 distal covariates and their effect on U5MR, time lags of zero and one years were applied for univariate regression, and zero, one, two and three years for multiple regression (**Table 2**).

**Table 2:** Unadjusted and adjusted association between U5MR and the social, economic, political and health system determinants, by time lag

Determinants	Univariate regression		Multivariate regression			
	**No lag**	**1 year lag**	**No lag**	**1 year lag**	**2 year lag**	**3 year lag**
Health system and policy factor						
Beta (95% CI)	−9.426 (−10.946, −7.905)‡	−9.415‡ (−10.952, −7.879)	3.555‡ (1.052, 6.059)	4.109‡ (1.448, 6.771)	4.511‡ (1.746, 7.276)	5.450‡ (2.565, 8.336)
Constant (95% CI)	30.061 (25.704, 34.419)‡	27.683‡ (23.533, 31.834)				
R^2^	0.252	0.256				
Health programmes and interventions factor						
Beta (95% CI)	−16.559 (−17.697, −15.421)‡	−13.936‡ (−14.994, −12.879)	−5.044‡ (−6.486, −3.602)	−3.452‡ (−4.778, −2.126)	−2.302‡ (−3.529, -1.075)	−1.228† (−2.377, −0.079)
Constant (95% CI)	30.061 (27.289, 32.834)‡	27.894‡ (25.273, 30.516)				
R^2^	0.660	0.649				
Economic factor						
Beta (95% CI)	−11.984‡ (−13.146, −10.822)	−11.773‡ (−12.908, −10.638)	−1.827 (−4.875, 1.221)	−2.084 (−5.357, 1.190)	−2.358 (−5.732, 1.017)	−3.059* (−6.494, 0.375)
Constant (95% CI)	30.061‡ (26.437, 33.686)	27.302‡ (23.803, 30.801)				
R^2^	0.466	0.468				
Social factor						
Beta (95% CI)	18.307‡ (17.247, 19.368)	16.304‡ (15.349, 17.259)	11.676‡ (9.642, 13.709)	11.787‡ (9.868, 13.705)	11.418‡ (9.626, 13.210)	11.470‡ (9.742, 13.199)
Constant (95% CI)	30.061‡ (27.600, 32.523)	27.534‡ (25.222, 29.846)				
R^2^	0.728	0.740				
Political factor						
Beta (95% CI)	17.566‡ (16.080, 19.051)	14.189‡ (12.843, 15.535)	6.013‡ (4.587, 7.440)	4.241‡ (2.930, 5.552)	3.071‡ (1.861, 4.280)	2.225‡ (1.090, 3.360)
Constant (95% CI)	30.061‡ (25.230, 34.893)	28.049‡ (23.488, 32.611)				
R^2^	0.315	0.276				
Constant						
			30.061‡ (27.820, 32.303)	27.663‡ (25.530, 29.795)	25.546‡ (23.512, 27.581)	23.708‡ (21.745, 25.671)
R^2^			0.786	0.782	0.778	0.777
No. of observations	510	480	510	480	450	420

U5MR – under-five mortality rate, CI – confidence interval

*P < 0.10.

†*P* < 0.05.

‡*P* < 0.01.

In an attempt to gain further programmatic insights from our data, the 30 provinces were stratified into two groups using three different criteria: (i) those above and below the median rate of U5MR decline (which was -1.720 per 1000 live births per year); (ii) those above and below the median U5MR in 1990 (which was 54.5 per 1000 live births); and (iii) those above and below the median GDP per capita in 2006 (which was US$ 1709). We conducted multivariate analyses (stratified analyses with 1-year time lag) of the 5 constructs separately in each subset of 15 provinces to identify the key determinants of child mortality reduction in different contexts (**Table 3**).

**Table 3:** Adjusted associations between U5MR and the social, economic, political, health system determinants with 1 year lag after stratifying by U5MR rate of decline, level of U5MR in 1990 and GDP per capita level in 2006

Determinants	U5MR rate of decline		U5MR in 1990		GDP per capita in2006	
	**≤median†**	**>median**	**≤median†**	**>median**	**≤median‡**	**>median**
Health system and policy factor						
Beta (95% CI)	6.784 (4.737, 8.831)‡	1.002 (−4.573, 6.578)	7.623‡ (5.521, 9.724)	−0.098 (−5.421, 5.226)	−1.954 (−8.415, 4.507)	5.499‡ (3.188, 7.809)
Health programmes and interventions factor						
Beta 95% (CI)	−2.852‡ (−4.107, −1.596)	−2.964‡ (−4.828, −1.101)	0.047 (−1.204, 1.298)	−3.820‡ (−5.668, −1.972)	−4.121‡ (−6.059, −2.184)	−1.06 (−2.529, 0.409)
Economic factor						
Beta (95% CI)	−6.640‡ (−9.373, −3.907)	−12.535‡ (−18.651, −6.418)	−8.293‡ (−11.145, −5.440)	−12.581‡ (−18.241, −6.920)	−9.400‡ (−15.657, −3.143)	−3.865† (−6.960, −0.770)
Social factor						
Beta (95% CI)	7.869‡ (6.046, 9.693)	6.120‡ (2.930, 9.310)	8.384‡ (6.706, 10.063)	5.610‡ (2.470, 8.750)	5.628‡ (2.359, 8.896)	11.901‡ (9.830, 13.972)
Political factor						
Beta (95% CI)	0.863 (−0.293, 2.020)	4.821‡ (2.837, 6.804)	0.955 (−0.463, 2.373)	3.639‡ (1.896, 5.382)	4.218‡ (2.220, 6.217)	2.307‡ (0.901, 3.713)
Constant						
Beta (95% CI)	23.108‡ (20.870, 25.347)	28.115‡ (24.797, 31.434)	21.731‡ (20.492, 22.969)	28.139‡ (24.889, 31.390)	27.166‡ (23.575, 30.757)	23.841‡ (21.480, 26.201)
R^2^	0.774	0.792	0.761	0.792	0.75	0.82
No. of observations	240	240	240	240	240	240

U5MR – under-five mortality rate, GDP – gross domestic product, CI – 95% confidence interval

*P < 0.10.

†*P* < 0.05.

‡*P* < 0.01.

§The median of the rate of decline was −1.720 per 1000 live births per year.

¶The median of U5MR in 1990 was 54.5 per 1000 live births.

||The median of GDP per capita in 2006 was US$ 1708.8 (2006 value).

## RESULTS

Between 1990 and 2006, the U5MR decreased substantially in all 30 provinces in China (**Figure 1**). It varied more than 9-fold across provinces in 1990, ranging from 13.7 to 126.7 per 1000 live births. In 2006 the variability in U5MR was still more than 8-fold, ranging from 4.81 to 38.9 per 1000 live births.

Among the crude and adjusted associations between the 35 indicators and U5MR based on 510 data points (ie, 30 provinces × 17 years) the strongest crude associations were observed for the indicator ‘hospital delivery rate” (R^2^ = 0.72), ‘crude birth rate” (R^2^ = 0.67), ‘child care systematic management rate” (R^2^ = 0.62), ‘household possession index” (R^2^ = 0.57) and ‘illiteracy rate of women aged 15+’ (R^2^ = 0.56) (Online Supplementary Document(Online Supplementary Document), table w4). In multivariate regression with all 35 determinants included, 88% of the overall variance in U5MR was explained by all 35 indicators (Online Supplementary Document(Online Supplementary Document), table w5).

Factor analysis showed that within each of the social, economic, political, health system and policy, and health programs and interventions constructs, all the indicators correlated well with the resulting factor (as suggested by the large factor loadings). Each of the 5 extracted factors captured 68–91% of the common within-group variation of its affiliated indicators (**Table 1**).

Crude and adjusted associations between U5MR and these 5 constructs are presented in **Table 2**. In the univariate analysis with one-year time lag, determinants within the social construct showed the strongest crude association with U5MR reduction (R^2^ = 0.74), followed by strong effects of health programmes and intervention (R^2^ = 0.65), economic determinants related to both population and local governments (R^2^ = 0.47), political determinants as measured by decentralization indices (R^2^ = 0.28) and health system and policy determinants (R^2^ = 0.26). When multivariate regression was applied with a one year time lag, 78% of the variation in the system was explained by the 5 constructs. Again, the social determinants showed the strongest effect (beta = 11.79, *P* < 0.0001), followed by political determinants (beta = 4.24, *P* < 0.0001), and health programmes and interventions determinants (beta = −3.45, *P* < 0.001). Health system and policy determinants had a counter-intuitive adjusted effect (beta = 4.11, *P* < 0.0025) and the effect of economic factor was not statistically significantly different from 0 (beta = −2.08, *P* = 0.2123) (**Table 2**).

The associations showed distinctive patterns of change when different time lags (0-3 years) were applied (**Table 2**). Social determinants were the only construct that did not seem sensitive to the time lag applied. The effects of health programmes and intervention determinants and political determinants diminished as the time lag increased from 0 to 3 years (beta values: −5.0, −3.4, −2.3, −1.2, and 6.0, 4.2, 3.1, 2.2, respectively). At the same time, the importance of health system and policy determinants and economic determinants increased steadily (beta values: 3.5, 4.1, 4.5, 5.5, and −1.8, −2.1, −2.4, −3.1, respectively). Our interpretation of this finding, which is important for health planning and resource allocation, is that social determinants of child survival act both within a short and mid-term period. The effects of health programmes, interventions and political budgetary decisions are more likely to be felt within a short time period. The effects of economic growth and investments into health systems also contribute substantially to child mortality reduction, but they require a mid-term period to be detected in full.

**Table 3** reports the results of the analyses stratified by U5MR decline, U5MR and GDP (as described above) with a 1 year time lag (the results for other studied time lags are shown in the Online Supplementary Document(Online Supplementary Document), table w6). The notable change was the increased importance of determinants in the health systems and policies construct in the sub-group of provinces that started with lower U5MR, higher GDP and slower declines in U5MR. With a few exceptions, the determinants in the social construct were nearly always associated with the largest contribution to U5MR reduction (beta range 5.6 to 11.9). Economic factors have a positive role in the reduction of child mortality across all 6 strata (beta range −3.9 to −12.6), followed by health programs and intervention determinants (beta range 0.1 to −4.1). However, the associations of determinants in the health program and intervention construct with U5MR differed across stratified groups. They seemed to have most importance in the 15 provinces with higher starting U5MR and lower GDP. The effect of political determinants was significant in the provinces with higher starting U5MR and faster rate of U5MR decline. In the 15 provinces with a faster-than-median rate of U5MR decline, economic determinants were the strongest factors independently associated with U5MR (one-year lag model beta = −12.5, *P* < 0.001), followed by social determinants (beta = 6.1, *P* < 0.001). The same pattern was observed in the study of association between the 5 constructs and U5MR in the 15 provinces with above-median baseline U5MR level (economic determinants: beta = −12.6, *P* < 0.001; social determinants: beta = 5.6; *P* < 0.001), and with below median levels of GDP per capita (economic determinants: beta = −12.6, *P* < 0.001; social determinants: beta = 5.6; *P* < 0.001) (**Table 3**).

We conducted several additional sensitivity analyses to examine the robustness of our reported results. We reclassified the ‘crude birth rate’ indicator from the social to health system and policy construct (Online Supplementary Document(Online Supplementary Document), table w7). Although this indicator increased the overall effect of the latter construct substantially across all 4 time lags, the construct with social indicators remained the most significant determinant of child survival reduction. This analysis gave 2 important results: (i) the large effect of social determinants on child survival reduction is not dependent on fertility reduction; and (ii) fertility reduction has a very strong independent effect on child mortality. We also repeated the multivariate analysis after excluding the indicators that were not associated with U5MR in the univariate analysis (Online Supplementary Document(Online Supplementary Document), table w8), again with little overall change to the main conclusions. Finally, we ran the analysis only using data for 1996–2006, to avoid any biases that may have been introduced by use of imputed trends in 16 provinces in the 1991−1995 period (Online Supplementary Document(Online Supplementary Document), table w9). None of these analyses generated substantially different results. We presented the immunization coverage for all main vaccines against childhood diseases in the 1990−2006 period, to demonstrate that vaccination rates remained consistently very high with little variation throughout the study period and were thus not expected to influence our results (Online Supplementary Document(Online Supplementary Document), table w10).

## DISCUSSION

We are not aware of any other studies of this scale that have explored the impact of many diverse determinants of child survival in large child populations over an extended period of time, during which genuine progress in U5MR reduction has been achieved. The results of our analysis showed that the identified determinants accounted for almost 90% of the observed U5MR reduction during the years examined.

### Importance of social determinants

The fall in U5MR observed in China since 1990 was most influenced by social determinants - although the health system, health program, political and economic determinants also had important and independent roles. Along with the creation of the community-based “barefoot doctor” health providers in rural areas (whose role also included also promotion of literacy, sanitation and hygiene), which was hailed as one of the foundations of the primary health care movement [30,31], the Chinese government launched effective efforts to control population growth even before the one-child policy. Those efforts had already halved the total fertility rate from 5.9 to 2.9 by 1979 [32,33]. Although good quality child mortality data are not available for China from 1950–1980, available data report a large reduction in infant mortality rate from about 250 per 1000 live births in 1950 to 50 by 1980 [34]. Based on our analysis, the continuing decline in China’s U5MR owes much to its broad social progress and political stability, with economic development also benefiting from these determinants, and in turn influencing the number of child deaths prevented [21,22,27,34].

### Importance of fertility decline

Our results suggest that China’s success in reducing fertility rates and the resulting community approaches to improved parenting and protection of child health had a major influence on child mortality. Although it is difficult to isolate this factor and make secure inferences about its independent effects, we found that fertility rate had the highest loading on the “social factor” cluster, which itself explained most of child mortality reduction. In these circumstances the effects of the other determinants that we studied may be attenuated in other countries in the absence of the level of fertility rate reduction observed in China. This hypothesis is reinforced by the sensitivity analysis presented in the Online Supplementary Document(Online Supplementary Document), table w7, where the indicator of fertility decline was moved to the health systems and policy construct where it substantially increased the effect size of this construct. There have been debates about the direction of the causal association between fertility reduction and child mortality reduction [35,36]. We believe that the example of China, where fertility was dramatically and suddenly reduced by law regardless of the second variable (U5MR), which then led to large reduction of U5MR during the following two decades, represents strong evidence in favor of a causal role of effective fertility measures on child mortality reduction.

### Variability of the impact of determinants of child mortality reduction

Social determinants seemed to be strongly associated with the reduction in U5MR when all 30 provinces, 35 indicators and 17 years were included in the analysis, closely followed by determinants in the health programmes and interventions construct. However, more detailed analyses revealed several interesting findings relevant for health policy and planning. If short-term effects are required, investments are better placed in social determinants, health programmes and interventions, and political determinants that include empowerment of local governments. However, if more strategic and long-term effects are expected, investments should once again support social determinants, but also health system development and economic development. In the context of a high baseline U5MR, low GDP and a planned rapid rate of U5MR decline, the greatest effect should be expected from action on economic and social determinants, but also health programmes and interventions and political determinants. However, in the context of low U5MR, higher GDP and a planned moderate rate of U5MR decline, the greatest effect should be expected from action on social determinants and health system and policy determinants. These findings are consistent with previous observations on similar data sets [15,18].

### Limitations of the study

There were many interesting potential determinants which we could not study in the absence of reliable year-to-year information. This includes immunization rates, although we performed a separate analysis of their likely effects on our overall results (Online Supplementary Document(Online Supplementary Document), table w10). We would have also liked to investigate the effects of more specific health-program variables (for example child nutrition status and practices, management of diarrhea and pneumonia, vitamin A supplementation), more detailed data on maternal education level, levels of health facility access and use, health insurance coverage, poverty thresholds, corruption indices, and many others [37-40]. None of these were included because we could not, at the time of analysis, obtain reliable information on any of these indicators from Chinese information sources. In this study, we used only indicators for which the available data during the period 1990–2006 suggested a level of completeness and reliability that would allow sufficient statistical power to address the main aims of this study. The Online Supplementary Document(Online Supplementary Document) shows the approaches and sensitivity analyses that we used to assure and verify the quality of our input data.

This Chinese example, in which child health inequities do not appear to have been widening over the past 15 years, is important as a case study in the wider global context [41,42]. We suggest there would be value in encouraging other nations to collate a similar set of determinants (for example through large scale intermittent surveys such as serial MICS and DHS augmented with data from other sources) and then apply the conceptual framework and methodology we adopt in this study. There have already been a few good reports of such analyses in the literature [15,43-45].

While we employed many excellent indicators to capture social, economic, health systems and policy, and health programmes and interventions determinants of U5MR reduction, it is very difficult to evaluate the impact of political determinants in the same way. We believe that our two political indicators represented a proxy of the level of decentralization and the spending power of the local governments. However, we believe that the mismatch between local resources and spending responsibilities in the absence of adequate central-local grants / transfers at the provincial and sub-provincial levels is an important political issue which may, in large part, explain why insufficient public resources are employed to target social and health indicators in poor localities [46]. Given the wide disparities within provinces, the provincial GDP per capita may have little impact on the living conditions (and U5MR) in remote ‘pockets of poverty’ within provinces. Future analyses should seek to extend and develop more appropriate indicators of political determinants to better reflect the well documented imbalance between available resources and spending responsibilities at the provincial and sub-provincial levels in China. Given the size of China’s provinces, such analyses will be highly relevant to similar analyses at country level elsewhere, and should contribute to reforms in the equity of public resource allocation.

### Conclusion

The results presented in this study support the recent calls to broaden vertical programs to include strengthening of health systems [47,48]. However our research suggests that this approach also has its limitations, as it potentially ignores the broader social, economic and political determinants that impact on all sectors of society. In addition to maternal and child health and nutrition programs, approaches to reducing child mortality should also incorporate improvements in general literacy and particularly education of women; access to fertility control options; access to clean water and sanitation; integration of minority populations, along with ensuring underlying political stability and good governance. As many of these determinants are not traditionally under the purview of health authorities, there is a risk that those determinants are inadequately considered in national approaches to reducing child mortality. An analysis of the relative importance of these and other determinants, if data are available, and the further study of the possible reasons for their impact, may help explain large disparities between the U5MRs of nations with similar rates of economic development. It may also explain the difficulty in further reducing U5MR after communicable disease mortality is controlled by disease-specific and other health- and nutrition-focused interventions. The WHO Commission on Social Determinants of Health was a step toward an analysis of these factors [49,50], but without convincing attempts until now to apply this approach to a key child health indicator such as U5MR.

In conclusion, this analysis has shown that China has achieved its remarkable progress in reducing U5MR through an inter-sectoral approach made possible through political stability over a prolonged period of time. The key characteristics of child mortality reduction were sustained economic growth and a focus on social development alongside key investments in health systems and expanded health intervention coverage.

## References

[1] Millennium Development Goals. Goal 4: Reduce Child Mortality. United Nations, New York, 2007. Available at: http://www.un.org/millenniumgoals/childhealth.shtml. Accessed: 2 May 2012.

[2] Bryce J, Daelmans B (2008). Countdown to 2015 for maternal, newborn, and child survival: the 2008 report on tracking coverage of interventions.. Lancet.

[3] Garrett E, Reid A (1995). Thinking of England and taking care: family building strategies and infant mortality in England and Wales, 1891-1911.. Int J Popul Geogr.

[4] Fulton JP (1980). Socioeconomic forces as determinants of childhood mortality decline in Rhode Island, 1860-1970: a comparison with England and Wales.. Comp Soc Res.

[5] Kunitz SJ (1983). Speculations on the European Mortality Decline.. Econ Hist Rev.

[6] Bideau A, Desjardins B, Brignoli HP. Infant mortality in the past. Oxford: Oxford University Press; 1997.

[7] McKeown T. The origins of human disease. Oxford: Blackwell Publishers; 1988.

[8] Preston SH (2003). The changing relation between mortality and level of economic development. 1975.. Bull World Health Organ.

[9] The World Bank. World Development Indicators. Washington DC: The World Bank; 2010. Available at: http://data.worldbank.org/indicator. Accessed: 2 May 2012.

[10] Gapminder. Data in Gapminder World. List of indicators. Available at: www.gapminder.org. Accessed: 2 May 2012.

[11] Moser KA, Leon DA, Gwatkin DR (2005). How does progress towards the U5MR millennium development goal affect inequalities between the poorest and least poor? Analysis of demographic and health survey data.. BMJ.

[12] Mogford L (2004). Structural determinants of U5MR in sub-Saharan Africa: A cross-national study of economic and social influences from 1970 to 1997.. Soc Biol.

[13] Barros FC, Victora CG, Scherpbier R, Gwatkin D (2010). Socioeconomic inequities in the health and nutrition of children in low/middle income countries.. Rev Saude Publica.

[14] Li J, Luo C, de Klerk N (2008). Trends in infant/U5MR and life expectancy in Indigenous populations in Yunnan Province, China.. Aust N Z J Public Health.

[15] Wang L (2003). Determinants of U5MR in LDCs: empirical findings from demographic and health surveys.. Health Policy.

[16] Gwatkin DR, Rutstein S, Johnson K, Suliman E, Wagstaff A, Amozou A. Socio economic differences in health, nutrition, and population within developing countries: an overview. Washington DC: World Bank;2007. 18293634

[17] Anand S, Bärnighausen T (2004). Human resources and health outcomes: cross-country econometric study.. Lancet.

[18] Houweling TAJ, Kunst AE, Looman CWN, Mackenbach JP (2005). Determinants of under-5 mortality among the poor and the rich: a cross-national analysis of 43 developing countries.. Int J Epidemiol.

[19] Victora CG, Barros FC, Huttly SR, Teixeira AM, Vaughan JP (1992). Early childhood mortality in a Brazilian cohort: the roles of birthweight and socioeconomic status.. Int J Epidemiol.

[20] Ohenjo N, Willis R, Jackson D, Nettleton C, Good K, Mugarura B (2006). Health of Indigenous people in Africa.. Lancet.

[21] National Bureau of Statistics China. China statistical yearbook 2007 [in Chinese]. Beijing: China Statistics Press; 2007.

[22] Zhuang YE, Zhang LP. Basic data on China population since 1990. Beijing: China Population Press; 2003.

[23] Rudan I, Chan KY, Zhang JSF, Theodoratou E, Feng XL, Salomon JA (2010). Causes of deaths in children younger than 5 years in China in 2008.. Lancet.

[24] Feng XL, Xu L, Guo Y, Ronsmans C (2011). Socioeconomic inequalities in hospital births in China between 1988 and 2008.. Bull World Health Organ.

[25] Feng XL, Guo S, Hipgrave D, Zhu J, Zhang L, Song L (2011). China's facility-based birth strategy and neonatal mortality: a population-based epidemiological study.. Lancet.

[26] Law on Maternal and Infant Health Care in China. Available at: http://www.unescap.org/esid/psis/population/database/poplaws/law_china/ch_record006.htm. Accessed: 2 May 2012.

[27] Department of Maternal and Child Health, Ministry of Health of the People’s Republic of China. Compilation Report for the Maternal and Child Health Policy Research [in Chinese]. Beijing: Department of Maternal and Child Health, Ministry of Health of the People’s Republic of China; 2006.

[28] Mosley WH, Chen LC (2003). An analytical framework for the study of child survival in developing countries. 1984.. Bull World Health Organ.

[29] Hill K (2003). Frameworks for studying the determinants of child survival.. Bull World Health Organ.

[30] Blumenthal D, Hsiao W (2005). Privatization and its discontents — The evolving Chinese health care system.. N Engl J Med.

[31] Lawn JE, Rohde J, Rifkin S, Were M, Paul VK, Chopra M (2008). Alma-Ata 30 years on: revolutionary, relevant, and time to revitalise.. Lancet.

[32] Hesketh T, Zhu WX (1997). The one child family policy: the good, the bad, and the ugly.. BMJ.

[33] Hesketh T, Xing ZW (2005). The effect of China’s one-child family policy after 25 years.. N Engl J Med.

[34] Ministry of Public Health. Chinese Health Statistics Digest. Beijing: Ministry of Public Health; 1995.

[35] Grundy E, Kravdal Ř (2008). Reproductive history and mortality in late middle age among Norwegian men and women.. Am J Epidemiol.

[36] Palloni A, Rafalimanana H (1999). The effects of infant mortality on fertility revisited: new evidence from Latin America.. Demography.

[37] Victora CG, Adair L, Fall C, Hallal PC, Martorell R, Richter L (2008). Maternal and child undernutrition: consequences for adult health and human capital.. Lancet.

[38] Victora CG, Wagstaff A, Schellenberg JA, Gwatkin D, Claeson M, Habicht JP (2003). Applying an equity lens to child health and mortality: more of the same is not enough.. Lancet.

[39] Bryce J, Terreri N, Victora CG, Mason E, Daelmans B, Bhutta ZA (2006). Countdown to 2015: tracking intervention coverage for child survival.. Lancet.

[40] Theodoratou E, Johnson S, Jhass A, Madhi SA, Clark A, Boschi-Pinto C (2010). The effect of Haemophilus influenzae type b and pneumococcal conjugate vaccines on childhood pneumonia incidence, severe morbidity and mortality.. Int J Epidemiol.

[41] Gwatkin DR, Bhuiya A, Victora CG (2004). Making health systems more equitable.. Lancet.

[42] Gwatkin DR, Rutstein S, Johnson K, Suliman E, Wagstaff A, Amozou A. Socio economic differences in health, nutrition, and population within developing countries: an overview. Washington DC: World Bank;2007. 18293634

[43] Masanja H, de Savigny D, Smithson P, Schellenberg J, John T, Mbuya C (2008). Child survival gains in Tanzania: analysis of data from demographic and health surveys.. Lancet.

[44] Shmueli A (2004). Population health and income inequality: new evidence from Israeli time-series analysis.. Int J Epidemiol.

[45] Gubhaju B, Streatfield K, Majumder AK (1991). Socioeconomic, demographic and environmental determinants of infant mortality in Nepal.. J Biosoc Sci.

[46] World Bank. China: Promoting Growth with Equity, Country Study, Report No. 24169-CHA, World Bank. Beijing: Tsinghua University Press; 2003.

[47] Samb B, Evans T, Dybul M, Atun R, Moatti JP, World Health Organization Maximizing Positive Synergies Collaborative Group (2009). An assessment of interactions between global health initiatives and country health systems.. Lancet.

[48] The World Bank. Good practices in health financing. Lessons from reforms in low and middle income countries. Washington DC: The World Bank; 2008. Available at: http://siteresources.worldbank.org/INTHSD/Resources/376278-1202320704235/GoodPracticesOverview.pdf. Accessed: 2 May 2012.

[49] Marmot M, Commission on Social Determinants of Health (2007). Achieving health equity: from root causes to fair outcomes.. Lancet.

[50] Barros FC, Victora CG, Scherpbier RW, Gwatkin D. Health and nutrition of children: equity and social determinants. In: Blas E, Kurup AS (Eds). Equity, social determinants and public health programmes. Geneva: World Health Organization; 2010. Available at: http://whqlibdoc.who.int/publications /2010/9789241563970_eng.pdf. Accessed: 2May 21, 2012.

