# Supplementary material for: Social, economic, political and health system and program determinants of child mortality reduction in China between 1990 and 2006: A systematic analysis
Source: J Glob Health. 2012 Jun;2(1):010405. doi: 10.7189/jogh.02.010405 (PMC3484751; doi:10.7189/jogh.02.010405)
Supplement: Online Supplementary Document [file jogh-02-010405-s001.pdf]

# Online Supplementary Document

**Table w1.** Description of the sources of the indicators

| |
|---|
| **Chinese Ministry of Health (MOH) – "China Health Statistics Yearbook"**<br><br>Every year, the Chinese MOH publishes the "China Health Statistics Yearbook (CHSY)" (the most recent version is for the year 2009). This document reports the Chinese government's official indicators related to health system and infrastructure, health personnel, facilities, rural and community health, maternal and child health, disease control and public health, registries and surveillance, food and water safety, environment and occupational health hazards and the basic demographic and socio-economic indicators. The CHSY reported child mortality data based on maternal and child health annual report system. We used the yearbook to obtain official estimates of under-five mortality rates for the 30 Chinese provinces from 1992 to 2006. |
| **Chinese Maternal and Child Health Annual Report System**<br><br>The annual report system is established as one of the four health statistics annual report systems in China. All county-level health departments are required to report on mandatory data to higher-level health departments. These data are then aggregated at provincial level and reported to MOH. In each county health department, one office is set up to manage the reporting system in their catchment area. Aggregated number of maternal and child deaths and live births are reported through this system. The system differs somewhat in urban and rural areas. In urban areas, all pregnancies are registered and community doctors keep a log of all pregnancy outcomes. When the pregnancy outcome is unknown, they call the woman's home to update the information. Once a month they also visit obstetric and emergency departments in the hospitals in their catchment area and check death certificates at the police departments to further identify maternal deaths. Community doctors send monthly reports to sub-district health managers, who then forward summary reports to district health managers twice a year. In rural areas, village doctors use their extensive community networks to identify births and deaths within their catchment area. Data are forwarded monthly to township hospitals and twice a year to the county health department (see also refs. 24 and 25 in the main text). |
| **Chinese National Report on neonatal, infant and under-five mortality**<br><br>For the starting year (1990) we obtained U5MR data for 30 provinces, measured as number of under-five deaths per 1,000 live births, from the Chinese national report on neonatal, infant and under-five mortality (see also refs. 10,22). We believe that those baseline rates are plausible because they were derived from a nation-wide neonatal, infant and under-five mortality rate study conducted in 1990 (22). The under-five mortality level for the year 1990 for each Chinese province, based on this source of data, is also available from the "Gapminder". The spreadsheet with the U5MRs for 1990 and 2000 from this report is available from: http://spreadsheets.google.com/pub?key=0AkBd6lyS3EmpcHA1OWFkUzNDSFdkelBVWW9CR3ZvVVE&gid=0 (Accessed: May 29, 2010) |
| **National and Provincial Statistics Yearbook** |

| |
|---|
| The Chinese Bureau of Statistics publishes China Statistical Yearbook annually. The Yearbook reflects the social and economic development in China. It presents annual data for key statistics in the past thirty years and covers some historically important years at the national and local levels of province, autonomous region and municipality directly under the central government. The Provincial Statistical Yearbooks serve a similar function, but at the province level. |
| **Health Finance Annual Report**<br><br>Chinese MOH also circulates a Health Finance Annual Report each year presenting provincial level health finance, amount of services delivered, and assets of public owned health care providers. Similar to the Maternal and Child Health Annual Report System, each county health department is responsible to aggregate these statistics from all public owned health care providers, who annually fills in and submits a national united health resource and health services report forms. |

**Table w2.** Description of analysis procedures and statistical modelling

| ***Steps*** | ***Tasks*** | ***Description*** |
|---|---|---|
| Step 1a – Under 5 mortality rate (U5MR) data | To obtain the official estimates of the U5MR for all 30 Chinese provinces for the years 1990 to 2006 | For the years 1992-2006 U5MR were obtained from the Chinese Health Statistics Yearbook (CHSY). The CHSY reported the provincial level estimates based on the Chinese maternal and child health annual report system (**Table w1)**. For the starting year (1990) we obtained U5MR data for 30 provinces, measured as number of under-five deaths per 1,000 live births, from the Chinese national report on neonatal, infant and under-five mortality (see also refs. 10,22). We then imputed the data in the period 1991-1995 in 16 provinces with the greatest level of concern over the quality of reported data and locally weighted smoothing (LOWESS) was applied to smooth U5MR in the imputed years. We based our decision on the simple set of criteria, including (1) the U5MR declined for more than 40% between 1990 and 1993; or (2) the U5MR declined more than 20% between 1990 and 1993, but it then increased for more than 5% between 1993 and 1996. In other 14 provinces, where we didn't have concern over the plausibility of the reported trend, the missing U5MR data for the year 1991 were imputed by assuming a linear trend between 1990 and 1992. The level and trend of U5MR for 1990-2006 for each province are presented in **Figure 1** in the main text. |
| Step 1b – Indicator data | To obtain the official estimates for 35 indicators of health | Official estimates for 35 indicators for each province and for the years 1990-2006 were obtained from the following sources: (1) National and Provincial Statistics Yearbook, (2) Chinese Health Statistics Yearbook and (3) Health Finance Annual Report. Details of these three sources are presented in **Table w1**. Details of all 35 indicators and their sources are presented in **Table w3**. |
| Step 2a – Univariate regression on indicators | To run a univariate regression for each of the 35 indicators | We run a univariate regression model to estimate the individual association between each indicator and U5MR. The type of model used was a random effect linear regression model, taking into account the clustering of annual U5MR within each province. Initially the unstandardized form of the indicators was used, and then a similar approach was applied after standardization of the indicators. The standardization was done by subtracting the mean of the indicators and divided by the standard deviation so that the standardized indicators have a mean 0 and standard deviation of 1. In **Table w4** the coefficients, p-values and $R^2$ values for all 70 univariate models (35 using the unstandardized form of the indicators and 35 using the standardized) are presented. |
| Step 2b - Multivariate regression on indicators | To run a multivariate regression with all the 35 indicators | A multivariate random effect linear regression model was applied with all 35 indicators (unstandardized and standardised form) entering the model. In **Table w5** the coefficients, p-values and $R^2$ values for the 2 multivariate models (1 using the |

| | | |
|---|---|---|
| | | standardized form of the indicators and 1 using the unstandardised) are presented. |
| Step 3 - Factor analysis | To create five factors for the five groups of indicators | The indicators were grouped into five different categories: (1) Social, (2) Economic, (3) Political, (4) Health system and policy, and (5) Health programmes and interventions (**Table w3**). For each of the five groups we run factor analysis (FA) and obtained five constructs, each one representing one group of variables, i.e. social, economic, political, and health system and policy, and health programmes and intervention constructs. The loading scores between each indicator and its construct are presented in **Table 1**. The decision on which indicators fall under which construct was made based on our "a priori" expectations, but it was changed if it became apparent that the greatest "loading" of each individual indicator is on some other major factor. We only needed to move 2 out of 35 factors "a posteriori" – urban and rural household crowding (from economic to social factor). |
| Step 4a – Univariate regression on constructs | To run a univariate regression for each of the five constructs | We run a univariate regression model to estimate the individual association between each construct and U5MR. The coefficients, constants, 95% confidence intervals and $R^2$ values for each univariate model are presented in **Table 2**. |
| Step 4b – Multivariate regression on constructs | To run a multivariate regression using the 5 constructs | The five constructs were entered into a multivariate random effect linear regression model, taking into account the clustering of annual U5MR within each province (**Table 2**). In total four multivariate regression models are presented: (a) 0 year lag, (b) 1 year lag, (c) 2 years lag and (4) 3 years lag (**Table 2**). |
| Step 5 – Stratified multivariate regression on constructs | To run a multivariate regression for each of the five constructs after stratification | Finally we run the multivariate random effect linear regression model of the five constructs after stratifying the 30 provinces according to their median (a) in rate of U5MR decline, (b) in level of U5MR in 1990, and (c) in level of GDP per capita in 2006. These 3 stratified analyses were conducted for no time lag (**Table w7**), 1 year time lag (**Table 3**), 2-year time lag (**Table w7**) and 3-year time lag (**Table w7**). |

**Table w3.** Description of the social, economic, political and health system determinants

**Social construct**

| Name | Acronym | Definition | Unit | Data source† |
|---|---|---|---|---|
| Percentage of ethnic minority autonomous counties | ethcounty | Ethnic minority county as a percentage of all counties in the corresponding provinces | % | 1 |
| Illiteracy rate of women aged 15+ | illit | Proportion of illiterate population aged 15+ out of total population 15+. Illiteracy is defined as people being able to read or write no or only little Chinese. Between 1991 and 1994, only literacy rate for both sexes combined are available. | | 1 |
| Crude birth rate | birthrate | No. of live births/ population *1000 | Per 1000 population | 1 |
| Urban household crowding | ucrowd | Inverse of the amount of living space per person | Person per square meters | 1 |
| Rural household crowding | rcrowd | Inverse of the amount of living space per person | Person per square meters | 1 |
| Percentage of household with clean water | cleanwater | Household level | % | 2 |
| Hygenic toilet coverage | toilet | Household level | % | 2 |
| Population density | popden | Population/square meters in each province | Population per square meters | 1 |

**Economic construct**

| Name | Acronym | Definition | Unit | Data source |
|---|---|---|---|---|

| | | | | |
|---|---|---|---|---|
| Urban household income | uinc | Disposable Income of urban households refers to the actual income at the disposal of members of the households which can be used for final consumption, other non-compulsory expenditure and savings. This equals to total income minus income tax, personal contribution to social security and subsidy for keeping diaries in being a sample household. The following formula is used: Disposable income = total household income - income tax - personal contribution to social security - subsidy for keeping diaries for a sampled household. | Yuan* per capita | 1 |
| Rural household income | rinc | Net Income refers to the total income of rural households from all sources minus all corresponding expenses. The formula for calculation is as follows: Net income = total income - taxes and fees paid - household operation expenses - taxes and fees depreciation of fixed assets for production - gifts to non-rural relatives. Net income is mainly used as input for reinvestment in production and as consumption expenditure of the year, and also used for savings and non-compulsory expenses of various forms. "Per capita net income of farmers" is the level of net income averaged by population, reflecting the average income level of rural households in a given area. | Yuan per capita | 1 |
| Urban living consumption | uexpen | Annual per capita consumption expenditure of urban households. Consumption expenditure of urban households refers to total expenditure of households for consumption in daily life, including expenditure on the eight categories of food; clothing; household appliances and services; health care and medical services; transport and communications; recreation, education and cultural services; housing; and miscellaneous goods and services. | Yuan per capita | 1 |
| Rural living consumption | rexpen | Annual per capita living expenditure of rural households. Total Expenditure refers to total expenses of rural households on production, consumption and redistribution, including expenditure on household operations; purchase of productive fixed assets; depreciation of productive fixed assets; taxes and fees; expenses on household consumption; expenses on properties; and expenses on transfers. | Yuan per capita | 1 |
| Household possessions index | possess | Possession of durable consumer goods. Based on the percentage of population in possession of TV, washer, motocycle or refrigerator in urban and rural areas, principle component analysis (PCA) was used to generate the household possession index with missing data imputed using regression for each province. | | 1 |

| | | | | |
|---|---|---|---|---|
| Bank savings per capita | saving_per | Reported by banks. | Yuan per capita | 1 |
| GDP per capita | gdp_per | | Yuan per capita | 1 |
| Electricity consumption per capita | electr_per | One indicator of general infrastructure | Kilowatt-hour | 1 |
| Paved roads per square kilometers | highway | Kilometers of paved roads divided by the total area of province | Kilometer per square kilometers | 1 |
| Cargo turnover per capita | hwturn | Freight traffic refers to the volume of freight transported with various means within a specific period of time. This indicator reflects the service of the transport industry towards the national economy and people's living conditions, as well as an important indicator used in formulating and monitoring transport production plans and research into the scale and pace of transport development. Freight transport is calculated in tons. Freight transport is calculated in terms of the actual weight of the goods and takes no account of the type of freight and distance of travel. | Tons per person | 1 |

**Political construct**

| Name | Acronym | Definition | Unit | Data source |
|---|---|---|---|---|
| % of local fixed assets investment | decen | It is calculated as urban investment in fixed assets as a percentage of total investment in fixed assets in the whole province. This indicator only has a value after 1993 when the taxation was decentralized. | % | 1 |
| Local tax revenue as % of GDP | taxasgdp | Index of decentralization or local financial capacity | % | 1 |

**Heath system and policy construct**

| Name | Acronym | Definition | Unit | Data source |
|---|---|---|---|---|

| | | | | |
|---|---|---|---|---|
| Number of health workers or doctors per 1000 population | heworker | | Per 1000 popualation | 2 |
| Number of hospital beds per 1000 population | hebed | | Per 1000 population | 2 |
| Outpatient medical costs per capita | opcost | | Yuan per capita | 3 |
| Inpatient medical costs per capita | ipcost | | Yuan per capita | 3 |
| Total spending on health per capita | heexpen | | Yuan per capita | 3 |
| Public spending on health per capita | pubhexpe | | Yuan per capita | 3 |
| Number of outpatient cases per 1,000 population | opvist | Level of health care seeking | | 3 |
| Fixed assets per capita | phasset | | Yuan per capita | 3 |
| Average salary of health workers | salary | | Yuan | 3 |

**Health programmes and interventions construct**

| Name | Acronym | Definition | Unit | Data source |
|---|---|---|---|---|
| Institutional delivery rate | hdr | | per 100 livebirths | 2 |
| Level of antenatal care | antevist | Percentage of pregnant women with 1+ antenatal visit divided by number of livebirths | Per 100 livebirths | 2 |
| Level of postpartum visit | postvist | 1+ visit | Per 100 livebirths | 2 |
| Level of neonatal visit by physician | neovist | 1+ visit | Per 100 livebirths | 2 |

| | | | | |
|---|---|---|---|---|
| Child care systematic management rate | sysman | Percentage of children under 3 who receive routine checkups, including growth and development checkups (anthropomertric checkups), routine blood tests and systematic physical checkups by pediatricians according to standard schedules (4:2:1 in urban areas and 3:2:1 in rural areas). | Per 100 children < 3 | 2 |
| Safe motherhood program | safemom | The program was initiated in 2000 in the following 11 high U5MR provinces: Sichuan, Tibet, Xinjiang, Yunnan, Ningxia, Gansu, Guizhou, Jiangxi, Hunan, Inner Mongolia, and Qinghai. The variable is a dummy variable. | | Feng, et al, 2010 |

† Data source: 1=China Statistical Yearbook, 2=China Health Statistics Yearbook, 3=China Health Finance Annual Report.
* Yuan, the Chinese dollar, is the unit of Renminbi, the Chinese currency.

**Table w4.** Univariate random effect linear regression model of the 34 indicators on U5MR: (a) using the unstandardized form of the indicators and (b) using the standardized form of the indicators (acronyms of variables are explained in Table w3).

| | Univariate regression (constant term not reported) | |
|---|---|---|
| | **(a) Unstandardized indicators** | **(b) Standardized indicators** |
| safemom | -21.454*** | -7.689*** |
| p value | (0.00000) | (0.00000) |
| R2 | 0.000 | 0.000 |
| heworker | 0.296 | 0.657 |
| p value | (0.67905) | (0.67905) |
| R2 | 0.099 | 0.099 |
| hebed | 1.238 | 1.497 |
| p value | (0.10124) | (0.10124) |
| R2 | 0.040 | 0.040 |
| opcost | -0.085*** | -8.048*** |
| p value | (0.00000) | (0.00000) |
| R2 | 0.241 | 0.241 |
| ipcost | -0.114*** | -9.118*** |
| p value | (0.00000) | (0.00000) |
| R2 | 0.302 | 0.302 |
| heexpen | -0.047*** | -9.069*** |
| p value | (0.00000) | (0.00000) |
| R2 | 0.268 | 0.268 |
| pubhexpe | -0.370*** | -8.751*** |
| p value | (0.00000) | (0.00000) |
| R2 | 0.043 | 0.043 |
| opvist | 0.090*** | 7.415*** |
| p value | (0.00000) | (0.00000) |
| R2 | 0.018 | 0.018 |
| phasset | -0.052*** | -8.195*** |
| p value | (0.00000) | (0.00000) |
| R2 | 0.250 | 0.250 |
| salary | -0.001*** | -9.729*** |
| p value | (0.00000) | (0.00000) |

| | | |
|---|---|---|
| R2 | 0.240 | 0.240 |
| hdr | -0.752*** | -17.736*** |
| p value | (0.00000) | (0.00000) |
| R2 | 0.715 | 0.715 |
| antevist | -0.544*** | -9.323*** |
| p value | (0.00000) | (0.00000) |
| R2 | 0.367 | 0.367 |
| postvist | -0.873*** | -12.352*** |
| p value | (0.00000) | (0.00000) |
| R2 | 0.409 | 0.409 |
| neovist | -0.871*** | -12.530*** |
| p value | (0.00000) | (0.00000) |
| R2 | 0.407 | 0.407 |
| sysman | -0.637*** | -15.642*** |
| p value | (0.00000) | (0.00000) |
| R2 | 0.623 | 0.623 |
| uinc | -0.003*** | -11.287*** |
| p value | (0.00000) | (0.00000) |
| R2 | 0.392 | 0.392 |
| rinc | -0.009*** | -12.287*** |
| p value | (0.00000) | (0.00000) |
| R2 | 0.487 | 0.487 |
| uexpen | -0.004*** | -11.077*** |
| p value | (0.00000) | (0.00000) |
| R2 | 0.351 | 0.351 |
| rexpen | -0.012*** | -12.005*** |
| p value | (0.00000) | (0.00000) |
| R2 | 0.422 | 0.422 |
| possess | -6.233*** | -13.311*** |
| p value | (0.00000) | (0.00000) |
| R2 | 0.566 | 0.566 |
| saving_per | -0.001*** | -8.412*** |
| p value | 0.000 | (0.00000) |
| R2 | 0.305 | 0.305 |

| | | |
|---|---|---|
| gdp_per | -0.001*** | -9.566*** |
| p value | (0.00000) | (0.00000) |
| R2 | 0.353 | 0.353 |
| electr_per | -0.012*** | -11.485*** |
| p value | (0.00000) | (0.00000) |
| R2 | 0.277 | 0.277 |
| highway | -0.004*** | -10.025*** |
| p value | (0.00000) | (0.00000) |
| R2 | 0.343 | 0.343 |
| hwturn | -2.839*** | -15.110*** |
| p value | (0.00000) | (0.00000) |
| R2 | 0.289 | 0.289 |
| ethcounty | 0.238*** | 9.568*** |
| p value | (0.00000) | (0.00000) |
| R2 | 0.240 | 0.240 |
| illit | 1.590*** | 20.926*** |
| p value | (0.00000) | (0.00000) |
| R2 | 0.557 | 0.557 |
| birthrate | 3.488*** | 16.829*** |
| p value | (0.00000) | (0.00000) |
| R2 | 0.668 | 0.668 |
| ucrowd | 238.746*** | 8.788*** |
| p value | (0.00000) | (0.00000) |
| R2 | 0.160 | 0.160 |
| rcrowd | 1443.561*** | 19.040*** |
| p value | (0.00000) | (0.00000) |
| R2 | 0.384 | 0.384 |
| cleanwater | -0.360*** | -8.325*** |
| p value | (0.00000) | (0.00000) |
| R2 | 0.366 | 0.366 |
| toilet | -0.459*** | -11.182*** |
| p value | (0.00000) | (0.00000) |
| R2 | 0.445 | 0.445 |
| popden | -0.022*** | -9.854*** |

| | | |
|---|---|---|
| p value | (0.00000) | (0.00000) |
| R2 | 0.174 | 0.174 |
| decen | -0.306*** | -10.575*** |
| p value | (0.00000) | (0.00000) |
| R2 | 0.321 | 0.321 |
| taxasgdp | 0.982*** | 6.694*** |
| p value | (0.00000) | (0.00000) |
| R2 | 0.129 | 0.129 |

* p-value<0.10, ** p-value<0.05, *** p-value<0.01

**Table w5.** Multivariate random effect linear regression model of the 34 indicators on U5MR: (a) using the unstandardized form of the indicators and (b) using the standardized form of the indicators (acronyms of variables are explained in Table w3).

**Multivariate regression (constant term not reported)**

| Indicator | (a) Unstandardized indicators | (b) Standardized Indicators |
|---|---|---|
| Safemom | 4.903*** | 1.757*** |
| p value | (0.00011) | (0.00011) |
| Heworker | -0.041 | -0.091 |
| p value | (0.90348) | (0.90348) |
| Hebed | -0.179 | -0.217 |
| p value | (0.63386) | (0.63386) |
| Opcost | 0.084 | 7.991 |
| p value | (0.33569) | (0.33569) |
| Ipcost | 0.102 | 8.104 |
| p value | (0.20184) | (0.20184) |
| Heexpen | -0.067 | -13.040 |
| p value | (0.37109) | (0.37109) |
| Pubhexpe | -0.053 | -1.258 |
| p value | (0.52232) | (0.52232) |
| Opvist | -0.024*** | -1.963*** |
| p value | (0.00216) | (0.00216) |
| phasset | -0.003 | -0.493 |
| p value | (0.77367) | (0.77367) |
| salary | -0.000 | -0.202 |
| p value | (0.89008) | (0.89008) |
| hdr | -0.248*** | -5.848*** |
| p value | (0.00000) | (0.00000) |
| antevist | -0.077** | -1.323** |
| p value | (0.03712) | (0.03712) |
| postvist | 0.125** | 1.766** |
| p value | (0.03873) | (0.03873) |
| neovist | -0.185*** | -2.666*** |
| p value | (0.00002) | (0.00002) |

| | | |
|---|---|---|
| sysman | -0.050** | -1.227** |
| p value | (0.04474) | (0.04474) |
| uinc | -0.002** | -6.687** |
| p value | (0.01824) | (0.01824) |
| rinc | -0.005*** | -7.272*** |
| p value | (0.00089) | (0.00089) |
| uexpen | -0.000 | -0.652 |
| p value | (0.63337) | (0.63337) |
| rexpen | 0.004** | 3.875** |
| p value | (0.03225) | (0.03225) |
| possess | 0.833 | 1.779 |
| p value | (0.14217) | (0.14217) |
| saving_per | -0.000 | -0.232 |
| p value | (0.89415) | (0.89415) |
| gdp_per | 0.001*** | 10.408*** |
| p value | (0.00001) | (0.00001) |
| electr_per | -0.003*** | -2.538*** |
| p value | (0.00063) | (0.00063) |
| highway | -0.000 | -0.514 |
| p value | (0.46832) | (0.46832) |
| hwturn | -0.225* | -1.199* |
| p value | (0.08009) | (0.08009) |
| ethcounty | 0.035*** | 1.412*** |
| p value | (0.00689) | (0.00689) |
| illit | 0.328*** | 4.324*** |
| p value | (0.00000) | (0.00000) |
| birthrate | 0.719*** | 3.468*** |
| p value | (0.00000) | (0.00000) |
| ucrowd | 26.653* | 0.981* |
| p value | (0.08409) | (0.08409) |
| rcrowd | -138.022** | -1.821** |
| p value | (0.01250) | (0.01250) |
| cleanwater | -0.018 | -0.419 |
| p value | (0.36317) | (0.36317) |

| | | |
|---|---|---|
| toilet | 0.017 | 0.419 |
| p value | (0.55394) | (0.55394) |
| popden | -0.003 | -1.183 |
| p value | (0.24438) | (0.24438) |
| decen | -0.065*** | -2.255*** |
| p value | (0.00022) | (0.00022) |
| taxasgdp | 0.325*** | 2.217*** |
| p value | (0.00000) | (0.00000) |
| Constant | 64.846*** | 30.061*** |
| p value | (0.00000) | (0.00000) |
| R2 | 0.882 | 0.882 |
| N | 510 | 510 |

* p-value<0.10, ** p-value<0.05, *** p-value<0.01

**Table w6.** Multivariate random effect linear regression model of the five constructs after stratifying by (a) U5MR rate of decline, (b) level of U5MR in 1990 and (c) GDP per capita level in 2006 for 0, 2 and 3 years of lag

**no lag**

| | U5MR rate of decline | | U5MR in 1990 | | GDP per capita in 2006 | |
|---|---|---|---|---|---|---|
| **Determinants** | **<=median⁺** | **>median** | **<=median†** | **>median** | **<=median‡** | **>median** |
| Health system and policy factor | 6.943*** | 0.353 | 6.677*** | -1.479 | -1.774 | 4.852*** |
| | [5.142,8.745] | [-4.859,5.564] | [4.732,8.622] | [-6.531,3.573] | [-7.601,4.053] | [2.584,7.120] |
| Health programmes and intervention factor | -3.800*** | -4.827*** | -1.135 | -5.667*** | -5.971*** | -2.249*** |
| | [-5.089,-2.511] | [-6.802,-2.852] | [-2.492,0.222] | [-7.672,-3.662] | [-7.983,-3.959] | [-3.926,-0.572] |
| economic factor | -7.314*** | -11.261*** | -7.203*** | -10.254*** | -9.184*** | -3.374** |
| | [-9.678,-4.950] | [-16.875,-5.646] | [-9.796,-4.611] | [-15.441,-5.067] | [-14.782,-3.585] | [-6.356,-0.392] |
| social factor | 7.517*** | 5.478*** | 8.884*** | 5.397*** | 4.498*** | 12.594*** |
| | [5.682,9.351] | [2.226,8.730] | [7.112,10.656] | [2.157,8.637] | [1.224,7.773] | [10.292,14.895] |
| political factor | 1.174* | 8.094*** | 1.883** | 6.276*** | 7.245*** | 2.669*** |
| | [-0.024,2.373] | [6.030,10.158] | [0.363,3.403] | [4.401,8.151] | [5.208,9.281] | [1.050,4.287] |
| Constant | 25.228*** | 31.466*** | 24.153*** | 31.475*** | 30.388*** | 26.413*** |
| | [22.910,27.546] | [28.100,34.833] | [22.929,25.377] | [28.175,34.776] | [26.775,34.002] | [23.958,28.867] |
| R2 | 0.778 | 0.818 | 0.768 | 0.811 | 0.777 | 0.818 |
| No. of observations | 255 | 255 | 255 | 255 | 255 | 255 |

**2 years lag**

| | U5MR rate of decline | | U5MR in 1990 | | GDP per capita in 2006 | |
|---|---|---|---|---|---|---|
| **Determinants** | **<=median⁺** | **>median** | **<=median†** | **>median** | **<=median‡** | **>median** |
| Health system and policy factor | 5.898*** | -0.062 | 7.277*** | -0.459 | -3.4 | 5.909*** |
| | [3.683,8.112] | [-6.013,5.890] | [5.149,9.405] | [-6.169,5.250] | [-10.224,3.424] | [3.600,8.219] |
| Health programmes and intervention factor | -1.962*** | -1.967** | 0.559 | -2.515*** | -2.701*** | -0.541 |
| | [-3.177,-0.748] | [-3.707,-0.227] | [-0.565,1.683] | [-4.231,-0.800] | [-4.546,-0.857] | [-1.835,0.753] |
| economic factor | -4.805*** | -13.872*** | -7.595*** | -15.060*** | -9.307*** | -4.141*** |
| | [-7.765,-1.844] | [-20.196,-7.548] | [-10.507,-4.684] | [-20.999,-9.121] | [-15.784,-2.830] | [-7.238,-1.043] |
| social factor | 8.629*** | 5.361*** | 8.202*** | 4.699*** | 6.059*** | 10.791*** |

| | | | | | | |
|---|---|---|---|---|---|---|
| | [6.861,10.396] | [2.243,8.479] | [6.675,9.728] | [1.640,7.759] | [2.852,9.265] | [8.960,12.622] |
| political factor | 1.162** | 2.326** | 0.752 | 1.651** | 2.145** | 2.164*** |
| | [0.046,2.278] | [0.440,4.213] | [-0.541,2.045] | [0.013,3.288] | [0.205,4.085] | [0.934,3.393] |
| Constant | 21.235*** | 24.719*** | 19.728*** | 24.678*** | 24.029*** | 21.588*** |
| | [19.094,23.376] | [21.290,28.148] | [18.484,20.971] | [21.368,27.989] | [20.368,27.691] | [19.284,23.893] |
| R2 | 0.771 | 0.754 | 0.757 | 0.751 | 0.722 | 0.814 |
| No. of observations | 225 | 225 | 225 | 225 | 225 | 225 |

**3 years lag**

| | **U5MR rate of decline** | | **U5MR in 1990** | | **GDP per capita in 2006** | |
|---|---|---|---|---|---|---|
| **Determinants** | **<=median⁺** | **>median** | **<=median†** | **>median** | **<=median‡** | **>median** |
| Health system and policy factor | 5.736*** | -0.576 | 6.892*** | 0.187 | -3.696 | 5.741*** |
| | [3.369,8.104] | [-7.239,6.088] | [4.781,9.003] | [-6.247,6.622] | [-11.425,4.033] | [3.387,8.095] |
| Health programmes and intervention factor | -1.435** | -0.624 | 0.284 | -0.481 | -0.859 | -0.651 |
| | [-2.645,-0.225] | [-2.222,0.974] | [-0.752,1.320] | [-2.063,1.101] | [-2.572,0.853] | [-1.868,0.565] |
| economic factor | -4.268*** | -13.949*** | -7.209*** | -16.399*** | -10.075*** | -3.698** |
| | [-7.395,-1.141] | [-20.280,-7.618] | [-10.088,-4.329] | [-22.440,-10.357] | [-16.663,-3.488] | [-6.824,-0.572] |
| social factor | 8.596*** | 6.178*** | 7.335*** | 6.026*** | 7.133*** | 9.993*** |
| | [6.828,10.363] | [3.191,9.164] | [5.902,8.767] | [3.098,8.954] | [4.072,10.194] | [8.242,11.744] |
| political factor | 0.788 | 1.227 | 0.016 | 0.781 | 1.196 | 1.583*** |
| | [-0.317,1.893] | [-0.534,2.988] | [-1.186,1.217] | [-0.744,2.305] | [-0.630,3.022] | [0.428,2.738] |
| Constant | 19.584*** | 21.958*** | 18.083*** | 21.754*** | 21.302*** | 19.877*** |
| | [17.552,21.616] | [18.343,25.573] | [16.847,19.318] | [18.318,25.191] | [17.474,25.131] | [17.610,22.145] |
| R2 | 0.764 | 0.729 | 0.753 | 0.732 | 0.713 | 0.801 |
| No. of observations | 210 | 210 | 210 | 210 | 210 | 210 |

⁺ The median of the rate of decline is -1.720 per 1,000 live births per year.

† The median of U5MR in 1990 is 54.5 per 1000 live births.

‡ The median of GDP per capita in 2006 is $ 1708.8 (2006 value).

* p-value<0.10, ** p-value<0.05, *** p-value<0.01

**Table w7.** Multivariate random effect linear regression model of the five constructs after regrouping general fertility rate from social into health system determinants

| Determinants | factor with 0 year lag | factor with 1 year lag | factor with 2 years lag | factor with 3 years lag |
|---|---|---|---|---|
| Health system and policy factor | 5.629*** | 6.262*** | 6.532*** | 7.735*** |
| | [2.956,8.302] | [3.453,9.070] | [3.639,9.424] | [4.711,10.760] |
| Health programmes and intervention factor | -6.081*** | -4.315*** | -3.008*** | -1.923*** |
| | [-7.531,-4.630] | [-5.659,-2.972] | [-4.255,-1.762] | [-3.107,-0.738] |
| economic factor | -4.439*** | -4.797*** | -4.985*** | -6.300*** |
| | [-7.574,-1.303] | [-8.133,-1.460] | [-8.382,-1.588] | [-9.765,-2.835] |
| social factor | 9.488*** | 9.896*** | 9.826*** | 9.584*** |
| | [7.471,11.505] | [8.001,11.791] | [8.079,11.573] | [7.895,11.272] |
| political factor | 6.192*** | 4.353*** | 3.130*** | 2.182*** |
| | [4.715,7.669] | [2.990,5.715] | [1.875,4.385] | [0.990,3.374] |
| Constant | 30.061*** | 27.630*** | 25.471*** | 23.591*** |
| | [27.673,32.449] | [25.349,29.910] | [23.287,27.656] | [21.464,25.718] |
| r2_o | 0.754 | 0.744 | 0.736 | 0.725 |
| N | 510 | 480 | 450 | 420 |

* p-value<0.10, ** p-value<0.05, *** p-value<0.01

**Table w8.** Multivariate random effect linear regression model of the five constructs after only including significant indicators from the univariate analysis

| **Determinants** | **factor with 0 year lag** | **factor with 1 year lag** | **factor with 2 years lag** | **factor with 3 years lag** |
|---|---|---|---|---|
| Health system and policy factor | 3.742*** | 4.256*** | 4.471*** | 5.249*** |
| | [1.289,6.195] | [1.658,6.854] | [1.772,7.169] | [2.435,8.063] |
| Health programmes and intervention factor | -5.012*** | -3.426*** | -2.295*** | -1.238** |
| | [-6.453,-3.570] | [-4.752,-2.099] | [-3.525,-1.065] | [-2.390,-0.086] |
| economic factor | -2.215 | -2.45 | -2.515 | -3.060* |
| | [-5.303,0.873] | [-5.758,0.858] | [-5.922,0.892] | [-6.523,0.402] |
| social factor | 11.687*** | 11.825*** | 11.517*** | 11.622*** |
| | [9.676,13.698] | [9.932,13.718] | [9.749,13.284] | [9.915,13.328] |
| political factor | 6.004*** | 4.237*** | 3.087*** | 2.266*** |
| | [4.585,7.424] | [2.934,5.539] | [1.886,4.289] | [1.139,3.393] |
| Constant | 30.061*** | 27.660*** | 25.548*** | 23.718*** |
| | [27.911,32.211] | [25.611,29.709] | [23.594,27.501] | [21.839,25.597] |
| r2_o | 0.791 | 0.788 | 0.785 | 0.785 |
| N | 510 | 480 | 450 | 420 |

* p-value<0.10, ** p-value<0.05, *** p-value<0.01

**Table w9.** Univariate and multivariate random effect linear regression model of the five constructs for the years 1996-2006

| | | **univariate regression no lag** | **univariate regression 1 year lag** | **factor with 0 year lag** | **factor with 1 year lag** | **factor with 2 years lag** | **factor with 3 years lag** |
|---|---|---|---|---|---|---|---|
| Health system and policy factor | | | | | | | |
| | beta | -5.623*** | -6.050*** | 2.907*** | 3.044** | 3.565** | 4.615** |
| | beta ci | [-6.605,-4.642] | [-7.219,-4.881] | [1.042,4.771] | [0.581,5.507] | [0.547,6.584] | [1.061,8.169] |
| | Constant | 24.018*** | 22.872*** | | | | |
| | Cons ci | [20.242,27.795] | [19.165,26.580] | | | | |
| | R2 | 0.225 | 0.221 | | | | |
| Health programmes and intervention factor | | | | | | | |
| | beta | -12.596*** | -11.725*** | -5.092*** | -2.862*** | -2.567*** | -3.373*** |
| | beta ci | [-14.042,-11.150] | [-13.243,-10.206] | [-6.512,-3.673] | [-4.406,-1.318] | [-4.175,-0.960] | [-4.950,-1.796] |
| | Constant | 26.830*** | 25.575*** | | | | |
| | Cons ci | [24.541,29.118] | [23.426,27.724] | | | | |
| | R2 | 0.653 | 0.663 | | | | |
| economic factor | | | | | | | |
| | beta | -7.270*** | -8.079*** | -5.414*** | -4.771*** | -5.298*** | -6.662*** |
| | beta ci | [-8.183,-6.357] | [-9.196,-6.961] | [-7.664,-3.165] | [-7.868,-1.673] | [-9.211,-1.386] | [-11.327,-1.996] |
| | Constant | 25.456*** | 24.225*** | | | | |
| | Cons ci | [22.249,28.663] | [21.055,27.394] | | | | |
| | R2 | 0.428 | 0.42 | | | | |
| social factor | | | | | | | |
| | beta | 11.532*** | 11.168*** | 6.485*** | 8.029*** | 7.831*** | 5.971*** |
| | beta ci | [10.643,12.421] | [10.289,12.047] | [5.037,7.934] | [6.450,9.608] | [6.223,9.439] | [4.350,7.591] |
| | Constant | 26.478*** | 25.100*** | | | | |
| | Cons ci | [24.486,28.469] | [23.134,27.065] | | | | |
| | R2 | 0.749 | 0.752 | | | | |
| political factor | | | | | | | |
| | beta | 22.538*** | 21.863*** | -0.731 | 1.124 | -1.553 | -0.1 |
| | beta ci | [17.125,27.950] | [16.602,27.124] | [-4.446,2.985] | [-2.771,5.019] | [-5.351,2.244] | [-3.722,3.522] |
| | Constant | 28.878*** | 27.819*** | | | | |
| | Cons ci | [24.403,33.354] | [23.429,32.210] | | | | |
| | R2 | 0.046 | 0.045 | | | | |

| | | | | | | |
|---|---|---|---|---|---|---|
| Constant | | | 27.702*** | 26.303*** | 23.968*** | 22.760*** |
| | | | [25.750,29.655] | [24.360,28.246] | [22.079,25.857] | [20.936,24.584] |
| R2 | | | 0.804 | 0.802 | 0.8 | 0.807 |
| No. of observations | 510 | 480 | 330 | 300 | 270 | 240 |

**Table w10.** WHO-UNICEF estimates of immunization coverage of China: 1990-2006

| **Vaccines** | 1990 | 1991 | 1992 | 1993 | 1994 | 1995 | 1996 | 1997 | 1998 | 1999 | 2000 | 2001 | 2002 | 2003 | 2004 | 2005 | 2006 |
|---|---|---|---|---|---|---|---|---|---|---|---|---|---|---|---|---|---|
| BCG *(Baccille Calmette Guérin vaccine)* | 99 | 97 | 94 | 92 | 89 | 79 | 84 | 83 | 83 | 85 | 85 | 85 | 84 | 85 | 86 | 86 | 92 |
| DTP1 *(First dose of diphtheria toxoid, tetanus toxoid and pertussis vaccine)* | 98 | 97 | 97 | 96 | 95 | 93 | 94 | 94 | 95 | 95 | 95 | 95 | 95 | 95 | 95 | 95 | 94 |
| DTP3 *(Third dose of diphtheria toxoid, tetanus toxoid and pertussis vaccine)* | 97 | 94 | 91 | 88 | 85 | 80 | 83 | 84 | 86 | 85 | 85 | 86 | 86 | 86 | 87 | 87 | 93 |
| MCV *(Measles-containing vaccine)* | 98 | 92 | 87 | 81 | 75 | 80 | 84 | 83 | 84 | 85 | 85 | 85 | 85 | 85 | 86 | 86 | 93 |
| Pol3 *(Third dose of polio vaccine)* | 98 | 95 | 93 | 90 | 87 | 82 | 84 | 85 | 86 | 86 | 86 | 86 | 86 | 86 | 87 | 87 | 94 |
